# Advances in Miniaturized Instruments for Genomics

**DOI:** 10.1155/2014/734675

**Published:** 2014-05-29

**Authors:** Cihun-Siyong Alex Gong, Kin Fong Lei

**Affiliations:** ^1^Department of Electrical Engineering, School of Electrical and Computer Engineering, College of Engineering, Chang Gung University, Taoyuan 333, Taiwan; ^2^Portable Energy System Group, Green Technology Research Center, College of Engineering, Chang Gung University, Taoyuan 333, Taiwan; ^3^Graduate Institute of Medical Mechatronics, Chang Gung University, Taoyuan 333, Taiwan; ^4^Department of Mechanical Engineering, Chang Gung University, Taoyuan 333, Taiwan

## Abstract

In recent years, a lot of demonstrations of the miniaturized instruments were reported for genomic applications. They provided the advantages of miniaturization, automation, sensitivity, and specificity for the development of point-of-care diagnostics. The aim of this paper is to report on recent developments on miniaturized instruments for genomic applications. Based on the mature development of microfabrication, microfluidic systems have been demonstrated for various genomic detections. Since one of the objectives of miniaturized instruments is for the development of point-of-care device, impedimetric detection is found to be a promising technique for this purpose. An in-depth discussion of the impedimetric circuits and systems will be included to provide total consideration of the miniaturized instruments and their potential application towards real-time portable imaging in the “-omics” era. The current excellent demonstrations suggest a solid foundation for the development of practical and widespread point-of-care genomic diagnostic devices.

## 1. Introduction

Genomics has become an important part of our life since its name was established in the latter half of the twentieth century. It was derived from genetics which includes “classic” and “molecular” as a whole. Polymer chain reaction (PCR) technique is a gold standard for clinical genomic diagnosis. Normally, the concentration of genomic sample is too low for generating detectable signal. PCR can amplify a few copies of DNA to millions of copies of a particular DNA sequence. The technique relies on thermal cycling, that is, repeated heating and cooling of the reaction, for DNA melting and enzymatic replication of the DNA. Generally, twenty to forty thermal cycle times are involved and they take several hours to complete. Although this technique is sensitive for genomic detection, it is time consuming and labor intensive, limiting the throughput of the diagnosis.

In order to enhance the efficiency of the biological reaction, reduce the usage of reagent and sample, and eliminate the fault by human handling, miniaturized instruments that handle small quantity of fluid, for example, microliter or nanoliter, were proposed for the next generation of the diagnostic equipment. Such instruments are also named as microfluidic systems, lab-on-chip (LOC) devices, biochips, or micrototal-analysis systems (*μ*TAS). Because fluid in small amount is manipulated in microscale environment, one of the important properties is to highly enhance the surface-to-volume ratio of the fluid. For some specific applications, high surface-to-volume ratio can benefit the process efficiency. For example, DNA hybridization in rapid diagnostic device normally involves a solid support for the immobilization of the reactants, that is, probe DNA strands. The counterpart of the reactant, that is, target DNA strands, is introduced to the site for binding reaction. The binding efficiency is based on the collision possibility. Because of the reduction in diffusion distance and increase in surface-to-volume ratio in microfluidic environment, the reaction kinetics of DNA strands binding reaction was shown significantly accelerated compared with the conventional microplate technique [1–5]. That results in greatly improving the response time of the biological reaction and the sensitivity of the biological detection. Microfluidic system is often interpreted to a miniaturized version of bioanalytical laboratory. It can perform the entire analytical protocol, such as sample preparation, reagent application, biological reaction, and detection automatically to eliminate the handling fault. Since microfluidic system is a miniaturized instrument, portability is realizable for the point-of-care diagnostic applications.

The aim of this paper is to report on recent developments on miniaturized instruments for genomic applications. An overview of microfluidic systems and their demonstrations for genomic diagnosis will be discussed. Moreover, impedimetric detection is found to be a promising technique for point-of-care genomic detection because the impedimetric signal can easily be analyzed by miniaturized electrical circuits. In-depth discussion of the consideration and review of impedimetric circuits and systems will also be included in this article.

## 2. Miniaturized Instruments: Microfluidic Systems

In the past decade, development of the microfluidic technology becomes intensive and many research articles are available [6–11]. The fabrication of microfluidic systems was originally based on the silicon fabrication technology from semiconductor and microelectromechanical systems (MEMS). Silicon microfabrication is well established but silicon material is not optically transparent and is electrically conductive. Hence, it is not appropriate for the biomedical applications. For example, the microfluidic system for cell culture is required to be transparent for continuous optical monitoring of cell morphology. Moreover, microfluidic system for glucose detection is based on electrochemical reaction which needs insulated substrate for measurement. Therefore, silicon may not be an appropriate material when optical and electrochemical detections are adopted in the microfluidic systems. Therefore, glass and polymeric materials were used because they are less expensive, optically transparent, and not electrically conductive. Specific fabrication technologies for microfluidic systems were introduced, such as soft lithography, hot embossing, and substrate bonding techniques. Soft lithography represents a nonphotolithographic strategy based on self-assembly and replica molding for carrying out micro- and nanofabrication [12]. An elastomeric stamp with patterned relief structures on its surface is used to generate patterns and structures with feature sizes ranging from 30 nm to 100 *μ*m. It provides a convenient, effective, and low-cost method for the formation and manufacturing of micro- and nanostructures. Hot embossing technique is for mass production of plastic microcomponents [13]. A mold with microstructures is pressed into a thermoplastic polymer film heated beyond its glass transition temperature under vacuum. After cooling down, the microstructures can be transferred from the mold to the polymer film. To fabricate a functional microfluidic system, substrate bonding is an important process and adhesion between substrates is a problem of great practical concern. Thermal compression, ultrasonic, or gluing by application of either epoxy or methanol may induce global and localized geometric deformation of the substrates or leave an interfacial layer with significant thickness variation. Therefore, special bonding processes for glass and polymeric materials have been developed for fabricating microfluidic systems [14–17]. Localized welding of polymeric materials embedded metal films located between the desired bond surfaces by microwave energy has been developed [15]. The bonding can be achieved with 10 W microwave power in 120 s.

**Figure 1:**
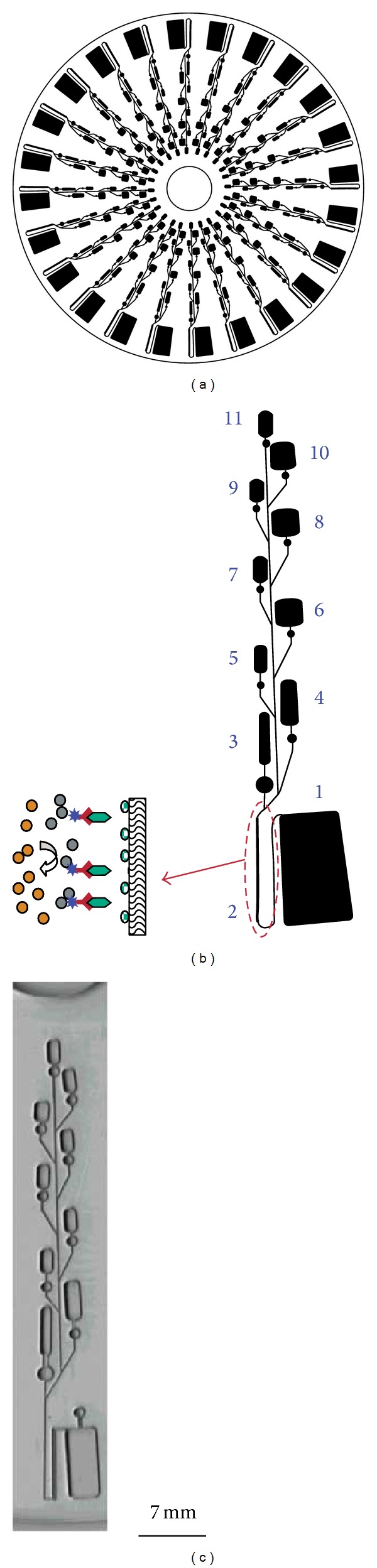
Schematics of (a) a CD-ELISA design with 24 sets of assays, (b) a single assay, and (c) photo of a single assay. Copyright 2004. Reprinted from [30] with permission from the American Chemistry Society.

**Figure 2:**
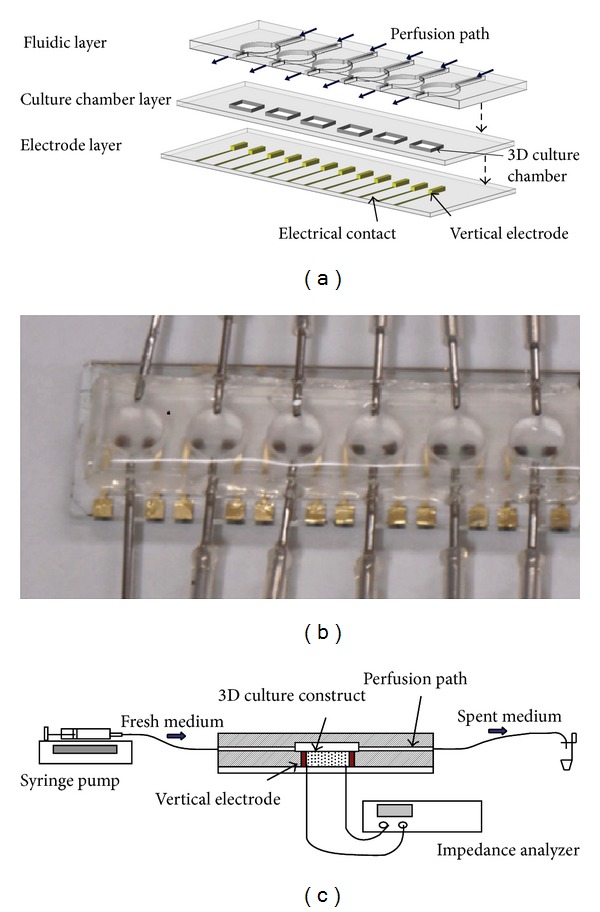
(a) Design of the microfluidic chip. (b) Photograph of the microfluidic chip. (c) Illustration of the experimental setup of the perfusion 3D cell culture incorporated with on-site impedance measurement. Copyright 2014. Reprinted from [37] with permission from the Elsevier.

Based on the mature development of the fabrication technology, a board spectrum of biological analytical applications has been demonstrated using microfluidic systems, such as DNA analysis [1, 18–24], immunoassay [25–31], and cell analysis [32–38]. For example, immunoassay on compact disc (CD) has been demonstrated and fluids in CD were manipulated by the centrifugal forces controlled by the rotational speed of the CD [30]. Illustration and photograph of the CD-based microfluidic system are shown in Figure 1. High throughput screening of analytes could be realized by simultaneous functions in parallel layouts on the CD. Enzyme-linked immunosorbent assay (ELISA) was demonstrated on this CD-based platform. Another example is to construct a microfluidic chip for real-time and noninvasive impedimetric monitoring of cell proliferation and chemosensitivity in three-dimensional (3D) cell culture construct, as shown in Figure 2 [37]. Human oral cancer cells (OEC-M1) were encapsulated in 3D agarose scaffold and cultured in a miniaturized chamber under perfusion of tested substance. This setting provides a more in vitro physiologically relevant microenvironment to better mimic the complex in vivo microenvironment. These excellent developments showed the capability of microfluidic system for performing complex analytical applications. Commercial possibility is obvious because the microfluidic system can provide a total solution of biological analysis from the sample application to the display of the analysis results. Point-of-care diagnostic applications can be realized based on the advantages of miniaturization, integration, and automation of the microfluidic system.

## 3. Integrated Microfluidic Genomic Systems

**Figure 3:**
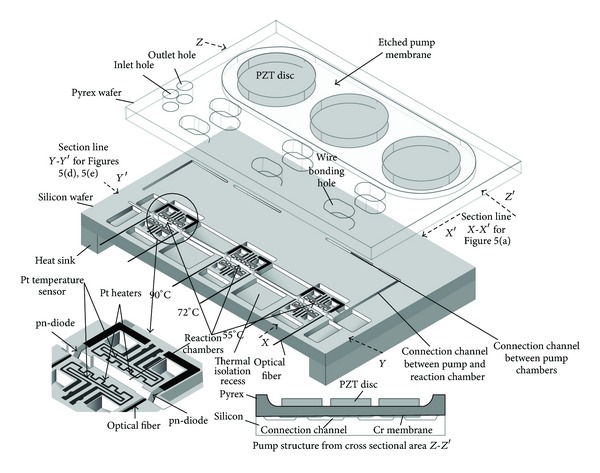
Schematic of the pump PCR chip. For simplification, the upper glass wafer and the lower silicon wafer are illustrated apart, although in the actual device both wafers are connected by anodic bonding. The lower left insert figure shows an expanded view of the reaction chamber and the lower right insert shows the cross-section of the micropump. Copyright 2003. Reprinted from [39] with permission from IOP Publishing Ltd.

**Figure 4:**
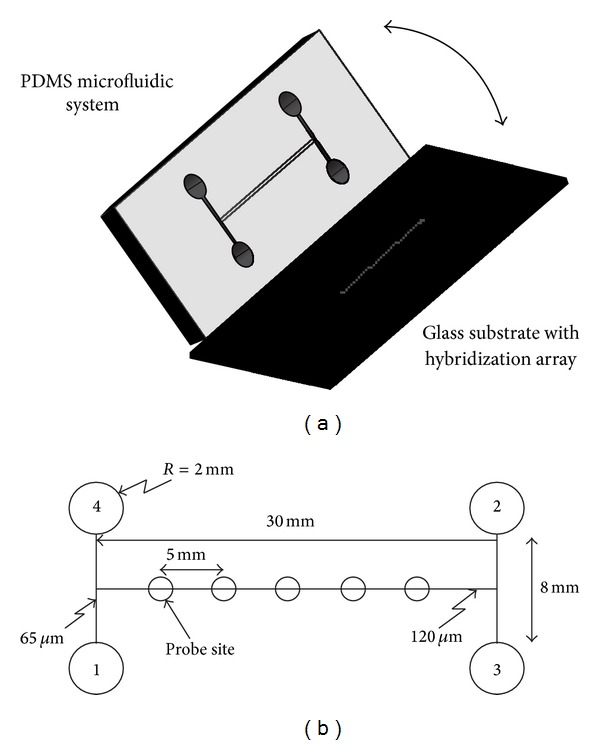
(a) Assembly procedure for PDMS fluidics and immobilized hybridization array. (b) H-type channel structure for DNA hybridization chip: (1) sample port, (2) auxiliary port, (3) buffer port, and (4) wash port. Copyright 2004. Reprinted from [1] with permission from the American Chemical Society.

Microfluidic systems have been also applied to the genomic applications. System integrated with microchannels, heaters, temperature sensors, and fluorescence detectors was fabricated for the functions of capturing DNA, mixing solutions, amplifying DNA, and separating and detecting of those products [20]. These complicated operations could be performed on a single glass and silicon substrate. Strand displacement amplification experiment was conducted and showed that the specific target DNA was successfully amplified and detected. Moreover, PCR is a widely used technique in biological applications and was implemented on a microfluidic system, as illustrated in Figure 3 [39]. The PCR was achieved by introducing the reactant droplet into the inlet. Three reaction chambers, respectively, stabled at 90°C, 72°C, and 55°C were integrated in a chip and droplet was driven back and forth by three piezoelectric micropumps between these three reaction chambers. After 20–30 thermal cycles, the PCR products were pumped into the reservoir to be collected and analyzed by gel electrophoresis. Also, an electrokinetically controlled DNA hybridization microfluidic chip has been demonstrated and can perform all processes from sample dispensing to hybridization detection within 5 minutes [1]. The chip consisted of a PDMS upper substrate and a lower glass substrate that served as a substrate for the hybridization array, as shown in Figure 4. The design of the chip was an H-type channel structure containing immobilized single-stranded oligonucleotide probes. The electroosmotic pumping could dispense the controlled samples of nanoliter volume directly to the hybridization array and remove nonspecific adsorption. Hybridization, washing, and scanning procedures can be conducted simultaneously. Detection levels as low as 50 pM were recorded using an epifluorescence microscope.

## 4. Impedimetric Detection of Genomic Signal

In conventional genomic detection, optical measurement, for example, fluorescent labeling technique, was utilized to quantify the genomic activity, for example, DNA hybridization and PCR product. But this measurement technique is time consuming and labor intensive. Alternatively, impedimetric detection was proposed to be one of the promising techniques to quantify biological activity in the microfluidic systems. The detection results are represented by electrical signals which can easily interface with miniaturized instruments. For example, electrical detection of DNA hybridization using electrochemical impedance spectroscopy (EIS) was demonstrated [40]. Results showed a 25% increase of impedance for double-stranded DNA on gold electrode compared with the same electrode with immobilized single-stranded DNA. Another example showed that the DNA hybridization could be detected by the resistance change across the electrode [41, 42]. DNA hybridization on a pair of electrodes was indicated by gold nanoparticles and the gold nanoparticles were physically amplified to a silver conductive layer on the electrode. The hybridization result could be measured by the conductivity changes across the electrode. These demonstrated showed an alternative method for detecting the genomic signal. For the application of cell proliferation study, the entire process requires a long period of time and in a controlled environment. It is more preferable to perform in a bench-top system. However, a miniaturized and portable device is more preferable for the on-site rapid diagnostic application. The combination of microfluidic and impedimetric technologies would be suitable for such a specific application.

## 5. Impedimetric Foundation

**Figure 5:**
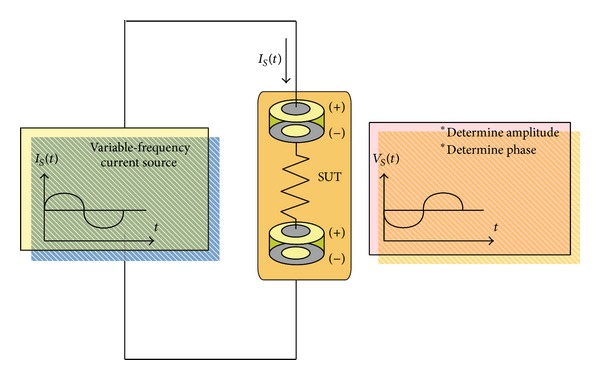
The basic model of equivalent circuit used to elaborate on the relation of sample under test (SUT) to the generated current source and resulting voltage response.

**Figure 6:**
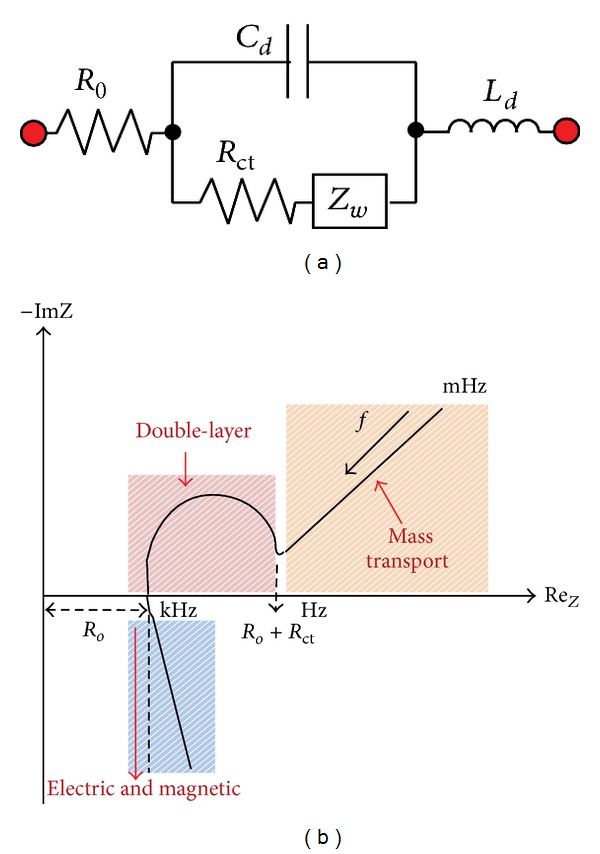
(a) Schematic of the equivalent circuit of electrode in an electrochemical point of view. (b) The so-called Nyquist plot showing the characteristic of frequency response versus the decomposition of impedance.

As mentioned, the impedimetric method provides a versatile way that can be used for many biological applications including the quantification of genomic activity and the noninvasive monitoring of cell proliferation and chemosensitivity with a microfluidic chip. The underlying principle of the technique can be explained from Figure 5. Let us assume, for the sake of simplicity, that the sample under test (SUT) consists only of a resistor that is connected with two electrodes. The upper electrode is commonly called “anode” or “working electrode.” The lower electrode is called “auxiliary electrode” functioning as “cathode.” To understand SUT, an active alternating current (AC), *I*_*S*_(*t*), is generated and injected into the close loop. The resulting voltage drop across the two electrodes can be measured to derive the resistance of SUT by means of Ohm's law, provided that both the two electrodes have zero voltage drop. When the equivalent circuit of electrode becomes a complex number, as the combination shown in Figure 6(a), variable-frequency current source is required to draw so-called “Nyquist plot” (Figure 6(b)) [43]. The model shown in Figure 6(a) is based on electrochemical point of view. It is consisted of an ohmic resistance *R*_*o*_ stemming from the solution resistance and electrode geometry, a charge transfer resistance *R*_ct_ stemming from the charge transfer between the interface of electrode and electrolyte, an electric double-layer capacitance *C*_*d*_ stemming from placing a large-area charge in the electrolyte in proximity to that on the porous electrodes at the medium-frequency region, a Warburg impedance *Z*_*w*_ representing the mobility of the internal ions resulting from the diffusion and migration at the low-frequency region, and an electrode inductance *L*_*d*_ stemming from reduction in the penetration depth of the ions at the high-frequency region, respectively [43, 44]. As a matter of fact, with regard to the ohmic resistance of SUT, an electrode immersed into an electrolyte creates a potential that is related to the oxidation-reduction concentration, according to the Nernst law [44]. The corresponding potentials cancel out as long as the two electrodes are the same. Unfortunately, this would never happen and a potential difference of a few millivolts would always exist between SUT and either of the electrodes [44].

## 6. Impedimetric Consideration

**Figure 7:**
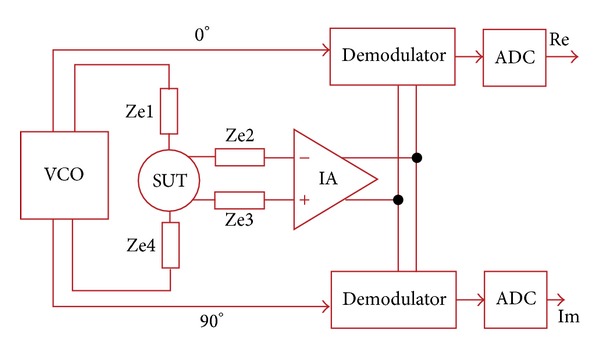
Impedance sensing architecture presented in [45], with which the four-electrode method accompanies, demonstrating the coherent demodulation technique.

There have been several technically sound circuits and systems demonstrated in the literature to implement the impedimetric method so far [44–49]. They are similar to a coherent demodulation technique demonstrated in Figure 7, where a four-electrode method was adopted [45]. The impedance sensing method shown in Figure 5 is premised on the assumption that both working and auxiliary electrodes have resistance value of “zero.” However, as mentioned, this would never be the case and there exist voltage drops of them in the close loop as soon as an electrical current flows through, turning out that certain inversion formula is unavoidable for derivation of the ohmic resistance of SUT. This may be taxing on postprocessing and result in incapability of real-time impedance monitoring. By taking the advantages of the advances in modern semiconductor technologies, an amplifier with ultrahigh input impedance (almost open circuit) can be readily available. In addition, differential sensing is always a better choice than the single-ended counterpart as a result of better noise immunity [46, 50]. These form the foundation of the architecture shown in Figure 7.

## 7. Circuits and Systems for Bioimpedance Measurement

Referring to Figure 7, in addition to the necessary electrodes Ze1 and Ze4 to form a loop, two additional electrodes Ze2 and Ze3 were added and combined with the instrumentation amplifier (IA) whose common-mode rejection ratio (CMRR) is significantly improved as compared with the ordinary counterpart [45, 46]. The variable-frequency sinusoidal current used for sensing was generated by a dedicated voltage-controlled oscillator (VCO). Thanks to the high-impedance feature of the amplifier, there was no current flowing through Ze2 and Ze3. As a result, the sensed voltage drop across SUT has predetermined current and therefore can be used to represent the impedance of SUT. A system with this kind of 4-electrode configuration is also known as a system using “tetrapolar method” [48].

**Figure 8:**
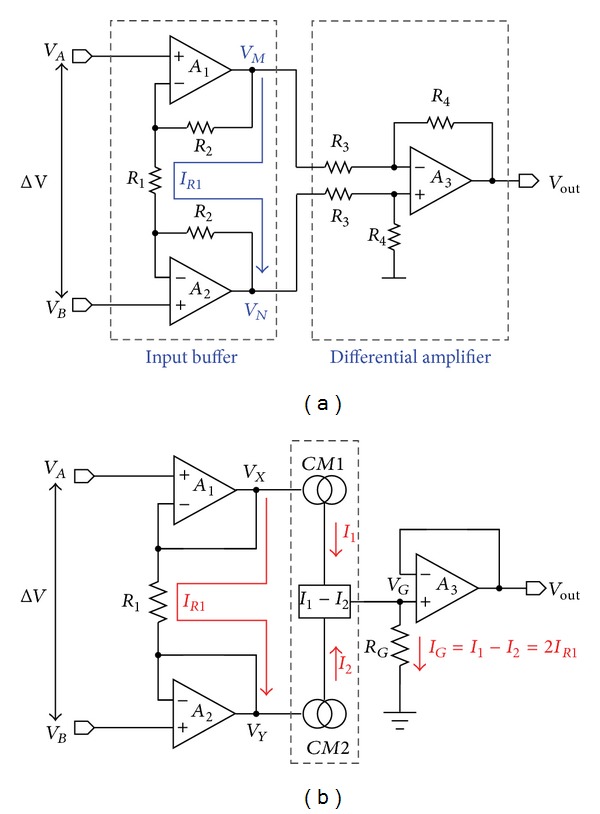
(a) The circuit schematic of conventional instrumentation amplifier (IA) in [50]. (b) The circuit schematic of improved counterpart in [51].

To decompose the complex number of impedance, two orthogonal AC signals are required in the coherent demodulation, based on the Euler's formula to represent a periodic signal using a combination of sine and cosine. The AC signals were generated in the same VCO to reduce the system complexity and save the implementation cost. The Demodulator circuits functioned as “mixer” and their outputs were quantized by the dedicated analog-to-digital converters (ADCs). The real part (Re) and imaginary part (Im) can be used to draw Nyquist plot for impedance analysis. It should be noticed that, practically, there still exists certain electric potential difference between Ze2 and Ze3 in spite of the zero current at the inputs of IA, and due to that Ze2 and Ze3 could not be identical. As a result, high CMRR is necessary to reject the potential difference of IA, resulting in design challenge. The major bottleneck in implementation is the matching of resistors involved in the commonly adopted IA structure shown in Figure 8(a) [50] where the output of IA can be expressed as

(1) $$\begin{array}{c} V_{\text{out}}=-\frac{R_{4}}{R_{3}}\left(1+\frac{2R_{2}}{R_{1}}\right)\left(V_{A}-V_{B}\right)=K_{1}\left(V_{A}-V_{B}\right). \end{array}$$

Achieving sufficient resistive matching between *R*_4_ and *R*_3_ (or *R*_2_ and *R*_1_) to obtain high CMRR relies on post-IC-fabrication trimming, which is cost ineffective and difficult to fulfill miniaturization in practice. As a result, the design shown in Figure 8(b) was proposed [51]. The most significant feature of the design is that it requires only two resistors. *A*_1_ and *A*_2_ form source followers as conventional, thereby forcing *V*_*A*_ = *V*_*X*_ and *V*_*B*_ = *V*_*Y*_. The current flowing out of *A*_1_ and that of *A*_2_ are equal, but they have opposite polarities. By using a current subtractor, marked in the dotted line, one can obtain *I*_*G*_ = 2*I*_*R*1_; hence the output of IA becomes

(2) $$\begin{array}{c} V_{\text{out}}=\frac{2R_{G}}{R_{1}}\left(V_{A}-V_{B}\right)=K_{2}\left(V_{A}-V_{B}\right). \end{array}$$

This circuit structure successfully alleviates the impact of mismatched resistance, achieving both high CMRR and miniaturization at the cost of increased power consumption as compared with that shown in Figure 8(a). High CMRR can also be attained by means of considerably increased differential gain. Unfortunately, the energy efficiency of system is further compromised.

**Figure 9:**
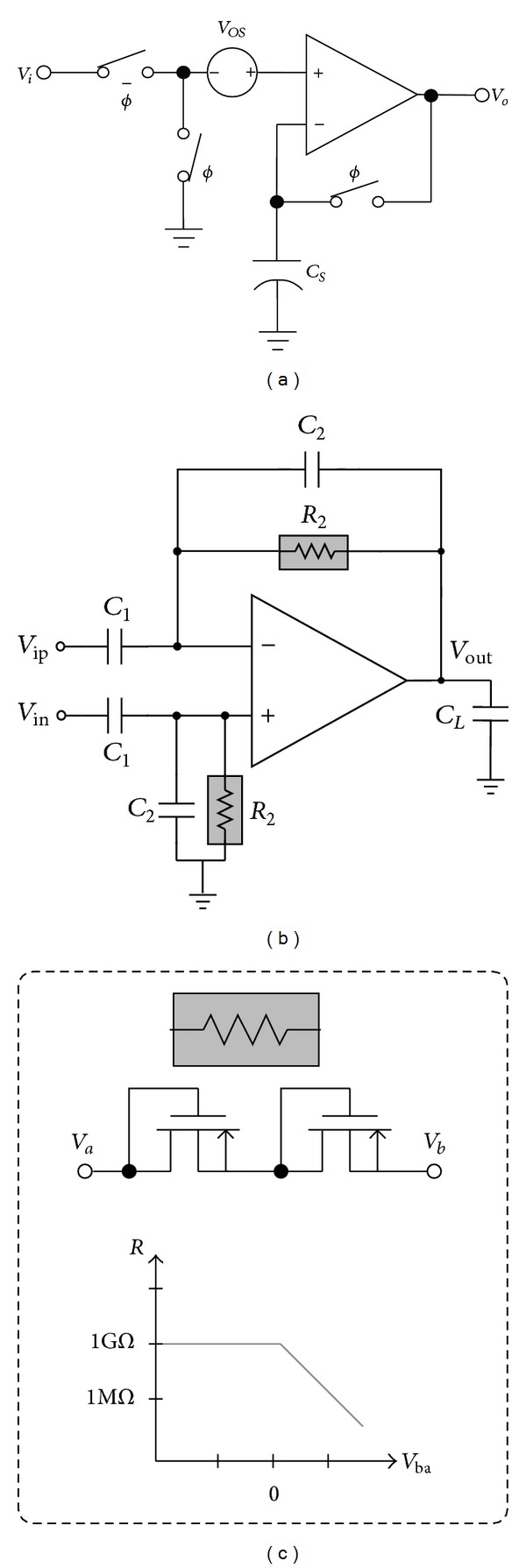
(a) The autozeroing technique [52]. (b) The AC-coupled technique [53]. (c) The pseudoresistor technique [53].

An often overlooked factor in correct impedance monitoring is that the electrode-referred DC offset (ERDO) limits the available CMRR, affecting the operation of IA and degrading overall performance no matter how good the following circuits and systems can be. Two renowned techniques have been proposed so far to cancel ERDO. A technique called “autozeroing” is shown in Figure 9(a) [52]. It uses three switches to cancel ERDO, *V*_*OS*_. When *ϕ* is at a logic-“high” level, the amplifier involved samples *V*_OS_ and store them on *C*_*S*_. Assuming that the open-loop gain of amplifier is *A*, the voltage on *C*_*S*_ will be *V*_*OS*_ · (*A*/(1 + *A*)) after settling. When *ϕ* becomes a logic-“low” level, *V*_*OS*_ of previous state will be subtracted from *V*_*i*_ which is superimposedwith current *V*_*OS*_ which will be with the same value as that of previous state, resulting in a considerably decreased ERDO of *V*_*OS*_ · (1/(1 + *A*)) present at the positive terminal of amplifier. The autozeroing technique can also effectively reduce the “Flicker” noise of modern semiconductor process but comes with a penalty of high-frequency interference stemming from the sampling clocks of the switches. Its major drawback is the wide bandwidth of amplifier as a result of the voltage settling on *C*_*S*_.

Another efficient candidate is the work shown in Figure 9(b) where *V*_ip_ and *V*_in_ can be connected with Ze2 and Ze3, respectively. The circuit serves as a preamplifier located between the electrodes and IA to “continuously” remove ERDO [53]. Here we use single differential circuit configuration to detail its advantage but then it can turn into its fully differential counterpart to provide two output terminals to IA. The design embodies the AC coupling to reject ERDO in order to make itself free from malfunction as a result of the saturation. The low-frequency cutoff of the high-pass filter formed by the *R*_2_-*C*_2_ network can be adjusted through their time constant. The low-pass corner frequency can be adjusted by the time constant of the lumped impedance at the output of the preamplifier and *C*_*L*_. Owing to the low frequencies required by the impedance measurement, an extremely large *R*_2_-*C*_2_ time constant is unavoidable. As a result, a pseudoresistor configuration shown in Figure 9(c) can be adopted [53]. The pseudoresistor operates the transistors involved at “subthreshold” operation to achieve a large equivalent resistance value that is almost impossible to be realized on the basis of “on-chip” miniaturization. The closed-loop midband gain of the preamplifier can be determined by *C*_1_/*C*_2_.

It should also be noticed that the accuracy of current of measurement, signals generated from VCO for the demodulation and how accurate their frequencies and phases can be achieved by the system shown in Figure 7, will also affect the final outcome dramatically. The frequencies and phases can be adjusted and finely tuned by means of a phase-locked loops with a precise reference frequency [54]. Such a frequency can be generated from an electronic circuit containing a mechanically resonant vibrating crystal (so-called crystal oscillator) [54]. Precise current of measuring SUT can be obtained through the use of a “current mirror” with sufficiently high output impedance. Modern semiconductor technologies offer many well developed and miniaturized circuit topologies to achieve such a goal [55, 56].

**Figure 10:**
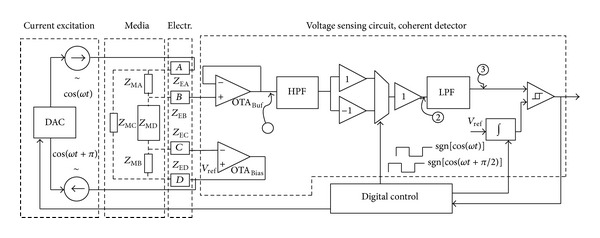
Synchronous sampling impedance sensing architecture. Copyright 2009. Reprinted from [48] with permission from Elsevier.

To advance miniaturization, the architecture shown in Figure 7 could be further improved as the complexity-reduced alternative shown in Figure 10 [48]. The architecture, which is called synchronous sampling, has mainly two most significant features: (a) removal of IA and (b) representing final results in pulse-width modulation (PWM) (using a one-bit ADC). The elements *Z*_EA_ to *Z*_ED_ correspond to the impedance of the four electrodes and the media were modeled by the elements *Z*_MA_ to *Z*_MD_. Each of these impedances has real and imaginary components associated with the conductivity and dielectric properties of the media, respectively [48]. The voltage on the negative terminal of OTA_BIAS_ will be forced to become the reference voltage *V*_ref_ which was set to halve the supply voltage and was used as the “ground” in the analog circuits involved, thanks to the high open-loop gains of the amplifiers achieving the “virtual short.” It turns out to be reducing the loss in the parasitic elements and avoiding the need for IA and the differential AC-coupled inputs. The demodulated results, followed by the low-pass filter (LPF), were compared with *V*_ref_ to obtain a PWM waveform that is easy to be transmitted wirelessly without parallel-serial converter commonly seen at the output of ADC for serial link. This architecture avoids two demodulation channels by incorporating a sampling mechanism using the proper sampling times.

**Figure 11:**
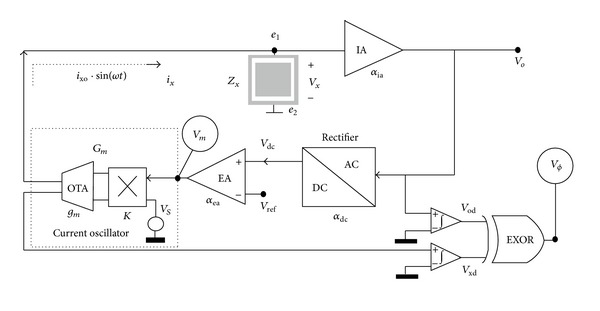
The closed-loop impedance sensing architecture. Copyright 2010. Reprinted from [49] with permission from Elsevier.

Recently, a closed-loop architecture shown in Figure 11 was proposed [49]. Despite the same theory principle for impedance measurement, its target used to determine the final outcome is unlike the two representative architectures shown in Figures 7 and 10. In this design, the resulting voltage across the impedance under test (ZUT) including SUT will be confined to a predetermined amount using the error amplifier EA with the given reference voltage *V*_ref_ at its input. This will help to operate the electrodes involved in a linear and predictable region. The generated AC current *i*_*X*_ flowing through ZUT can be controlled timely as a result of the feedback loop at *V*_*O*_. Because the transconductance of OTA (*g*_*m*_) can be deduced during design and measurement phases and both the multiplication factor *K* and signal source *V*_*S*_ can be given, *i*_*X*_ can be obtained, provided that *V*_*m*_ is available after being monitored. With *V*_*X*_ and *i*_*X*_, the impedance “magnitude” of ZUT can be measured. The impedance “phase” can be measured by comparing the digitized results *V*_od_ and *V*_xd_ of *V*_*O*_ and the output of OTA to each other.

Although the architecture shown in Figure 11 provides a good candidate to achieve not only an operation taking the contribution of electrodes into account but also a safe measurement with a decent accuracy as compared with others demonstrated in the literature, its overall performance is governed by the bandwidth, open-loop gain, input offset, and CMRR of amplifier, similar to its counterparts. However, high-gain, wide-bandwidth, low-offset, and high-CMRR amplifier consumes considerable power consumption, which goes against portability requiring miniaturization. In a nutshell, it has been believed that the performance of analog front end is of primary importance for the precise measurement of impedimetric system. The miniaturization effort involves making trade-offs among different aspects of mixed-signal (analog and digital) circuit design. The technical strategies illustrated with Figures 8 and 9 are by no means the total solutions but have demonstrated that they can be used to effectively deal with the mentioned problems in terms of miniaturization point of view. Last but not least, with regard to some implantable applications where an extremely miniaturized design of real-time impedance monitoring must be fulfilled in limited space to allowing integration to the most degree, the test current of SUT and ZUT could be generated by an electrical stimulator without the dedicated circuit such as VCO, DAC, or current oscillator shown in Figures 7, 10, and 11, respectively [56, 58, 59]. This turns out to be good for the system on a chip (SoC) in modern semiconductor technologies.

## 8. Impedimetric Imaging Instrumentation in Omics

**Figure 12:**
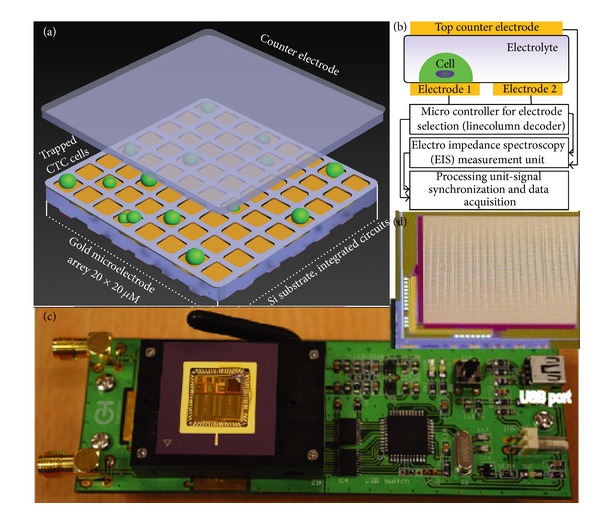
CMOS based sensor array for cell counting. (a) Schematic of the microelectrode arrays for the cell detection. (b) Illustration of the sensor layout and the addressing scheme employed in the CMOS sensor chip. (c) CMOS chip packaged with a switching PCB. (d) Microphotograph of more than nine thousand electrodes in a single chip. Copyright 2012. Reprinted from [57] with permission from Elsevier.

We have reviewed in detail the technologies regarding miniaturization. One might want to know the relevance between them and “imaging.” For the delivery of next-generation therapies, functional characterization of genes using a systematic way is imperative. One of the manners doing this kind of characterization requires downregulation of the expression of specific genes in order to comprehensively study the functions of genes [60]. To this end, the cell-based functional assay has been emerged as one of the powerful tools for acute observation [61]. The cell-based functional assay can be used to acquire the information about the phenotypic effect of targeted “gene knockdown,” which is a technique to reduce the expression of one or more of an organism's genes, in a way “incisive” when using RNA interference (RNAi) [62]. However, almost all of the assays are used only for experiencing a rapid onset (i.e., to say “for a given point of time”) currently, implying that most of the changes are missed in measurement. In addition, it has been demonstrated that advanced state-of-the-art electronic biosensors with microwell plates should be developed to be able to record impedimetric cell-to-electrode responses in a way “label-free” by means of microelectrodes. The combination of the requirements “continuous monitoring,” “impedimetric cell-to-electrode recording,” and “bidimensional-space (2D) electrode array manipulation” form the base of new-generation time-dependent profiling for cell responses. One of the most recent works with respect to the development of impedimetric spectrum platform with different application regarding cell has been demonstrated in Figure 12 where advanced semiconductor process and circuit techniques have been employed to advance miniaturized sensing system with light weight and low power in the platform [57]. By displaying the results of 2D spectrum continuously, real-time impedimetric imaging can be realized to ceaselessly measure the cell status of importance.

## 9. Concluding Remarks

The ever increasing demand in the modern technologies has improved the quality of life. Microfluidic systems have been applied to different genomic applications and showed realizable opportunity for point-of-care diagnostic devices. The advances in circuits and systems have been driving a technical revolution in the microfluidic systems that are essential to the “-omics” era. Impedimetric detection is a promising technique to develop miniaturized measurement equipment. The improved impact on the SoC techniques has enabled sustainable solutions which have been demonstrated so far to be effective to pressing real problems in such a field. Several representative solutions ranging from impedimetric architectures and efficiency-enhanced miniaturized techniques have been discussed in detail in this paper. Many researchers have pursued the ideas of using the techniques they have learned to facilitate interdisciplinary collaborations among SoC design, micromechanical technologies, material science, and biomedical engineering. It is almost impossible to embody miniaturization towards light weight and low power, while at the same time achieving an accurate impedance signal conditioning and the reduced response time without the help of microfabricated and mixed-signal technologies, not to mention the portability. In addition to the applications and advantages mentioned, it can be envisioned that by leveraging the architectures and techniques, low-price and precise early detection of many fatal diseases, such as the cancers, will eventually come true. Despite the strength and importance of impedance measurement system, those prior arts suffer the most from the contamination of electrode. Once the electrodes dip into the sample, nonspecific adsorption of biological components starts to take place. The contamination of electrode is still an open question and is accompanied with distortion of measured impedance spectrum, resulting in observable (inductive) artifact at some frequencies. In order to eliminate the contamination of detection electrodes and reaction chamber, the device is normally designed to be disposable for the rapid diagnostic applications. Moreover, the electrodes are made of noble metals, for example, Au and Pt, in order to prevent the surface oxidation. The contamination may also be overcome by having a large number of in vitro tests on electrode-sample reactions (redox) as an index of lookup stored in an on-chip memory. This may greatly help differentiate the shifted impedance spectrum from its normal circumstances (through some kinds of algorithms). In conclusion, the microfluidic systems incorporated with impedimetric detection technique provide simple, miniaturized, and sensitive detection of genomic signal. It is believed that these systems can develop practical point-of-care genomic diagnostic devices.
